# Preparation and Characterization of a Novel Decellularized Fibrocartilage “Book” Scaffold for Use in Tissue Engineering

**DOI:** 10.1371/journal.pone.0144240

**Published:** 2015-12-04

**Authors:** Liyun Guo, Jin Qu, Cheng Zheng, Yong Cao, Tao Zhang, Hongbin Lu, Jianzhong Hu

**Affiliations:** 1 Department of Sports Medicine, Research Centre of Sports Medicine, Xiangya Hospital, Central South University, Changsha Hunan 410008, China; 2 Department of Rehabilitation Medicine, Peace Hospital Attached to Changzhi Medical College, Changzhi Shanxi 046000, China; 3 Department of Orthopaedics, Hospital of Wuhan Sports University, Wuhan Hubei 430079, China; 4 Department of Spine Surgery, Xiangya Hospital, Central South University, Changsha Hunan 410008, China; Mayo Clinic Minnesota, UNITED STATES

## Abstract

At the tendon-to-bone insertion, there is a unique transitional structure: tendon, non-calcified fibrocartilage, calcified fibrocartilage, and bone. The reconstruction of this special graded structure after defects or damage is an important but challenging task in orthopedics. In particular, reconstruction of the fibrocartilage zone has yet to be successfully achieved. In this study, the development of a novel book-shape scaffold derived from the extracellular matrix of fibrocartilage was reported. Specifically, fibrocartilage from the pubic symphysis was obtained from rabbits and sliced into the shape of a book (dimensions: 10 mm × 3 mm × 1 mm) with 10 layers, each layer (akin to a page of a book) with a thickness of 100-μm. These fibrocartilage “book” scaffolds were decellularized using sequentially 3 freeze-thaw cycles, 0.1% Triton X-100 with 1.5 M KCl, 0.25% trypsin, and a nuclease. Histology and DNA quantification analysis confirmed substantial removal of cells from the fibrocartilage scaffolds. Furthermore, the quantities of DNA, collagen, and glycosaminoglycan in the fibrocartilage were markedly reduced following decellularization. Scanning electron microscopy confirmed that the intrinsic ultrastructure of the fibrocartilage tissue was well preserved. Therefore, the results of this study suggest that the novel “book” fibrocartilage scaffold could have potential applications in tissue engineering.

## Introduction

At the tendon-to-bone insertion, there is a physiologically unique transitional fibrocartilage zone, with calcified collagen fibers connecting to the bone and non-calcified collagen fibers connecting to the tendon. This graded transitional construction is formed under physiological loading conditions and is closely related to its mechanical properties [1–3]. Reconstruction of the tendon-to-bone insertion following defects or damage is a challenge for orthopedic surgeons. Previously, treatment involved using a suspending device to fix the tendon or ligament to the bone through surgical operations or using biodegradable interference fit fixation. Despite appropriate surgical management during such treatment, the original tendon—bone architecture may not be regenerated where fibrovascular scars have been formed. Because of incomplete regeneration of the original characteristic fibrocartilage zone between the tendon and bone, it is possible that the resultant tendon—bone junction may not be able to transfer load efficiently between the soft and hard tissues, which can lead to re-tearing or failure to heal [4–6]. Recently, growth factors and cytokines, physical stimulation, and stem cell transplantation have been used at the repair site with the aim of improving tendon-to-bone healing. However, to date, the graded transitional structure has yet to be successfully reconstructed [6, 7].

The development of tissue engineering technology, which often involves transplanting cells into synthetic or natural polymer scaffolds, has enhanced the study of tendon-to-bone interface regeneration. For example, Lu et al.[8] developed a three-phase poly(lactic-*co*-glycolic acid)/extracellular matrix (ECM) scaffold, with implanted fibroblasts, chondrocytes, and osteoblasts, which mimicked the transitional structure of the tendon-to-bone insertion. Distinct mineral and fibrocartilage-like tissue regions were formed on the scaffold, and collagen type II and aggrecan were detected. Chen et al.[9] developed a silk-RADA peptide scaffold, with implanted bone marrow stromal cells used to regenerate the cartilage—subchondral bone interface. They found that hypertrophic chondrocytes were embedded in the calcified ECM and that glycosaminoglycans (GAGs) and collagen were expressed. Despite some success, the synthetic biomaterials that were used in these studies could not fully simulate the original structure and matrix components of tendon-to-bone insertion. The anisotropy properties of the fibrocartilage zone at the tendon-to-bone insertion are closely related to its mechanical bearing capabilities and hence the successful reconstruction of the original structure of the fibrocartilage zone is particularly important.

Several studies have reported the use of decellularized cartilage in tissue engineering for articular cartilage reconstruction [10–12]. It is believed that a decellularized cartilage may be a viable option as a replacement, as the antigenic cellular material will be removed while preserving the relatively non-immunogenic extracellular matrix [13]. However, it is difficult to achieve cell migration into acellular cartilage scaffolds because they are highly dense with limited space [14]. Cartilage sheets and cartilage particles are often used in cartilage tissue engineering. For example, Gong et al. developed a “sandwich model” with 10- and 30-μm acellular cartilage sheets [11], whereas Yang et al. developed a 3D porous acellular scaffold by using acellular cartilage particles with diameters ranging from 500 nm to 5 μm [15]. Although these scaffolds were multi-aperture and cells could migrate into the acellular cartilage scaffold by a series of treatments, the original structure of the cartilage matrix was destroyed. Furthermore, a thin single-layer tissue chip is difficult to be operated in polyphase integration.

In this study, a decellularized fibrocartilage with a “book” design was developed for use in tissue engineering. The characteristics of the “book” scaffold were investigated by using various techniques: histology, to detect cellular components and structures; scanning electron microscopy, to determine the ultrastructure; and quantification assays, to measure levels of DNA, collagen, and GAG.

## Materials and Methods

### Experimental animals

Samples of fibrocartilage of the pubic symphysis were collected from 20 New Zealand white rabbits (18 weeks old, 2–3 kg), supplied by the Experimental Animal Center of Central South University. All rabbits were maintained under specific pathogen free conditions and fed standard rodent chow ad libitum. All experimental procedures conformed to the Chinese National Health and were approved by the Ethics Committee of the Center for Scientific Research with Animal Models of Central South University (2013-3-13). The pubic symphysis fibrocartilage samples were harvested from 20 New Zealand white rabbits which were euthanized (overdose of sodium pentobarbital, 100 mg/kg). None surgery was performed before euthanization. All efforts were made to minimize suffering.

### Preparation of “book” cartilage

**Fig 1:**
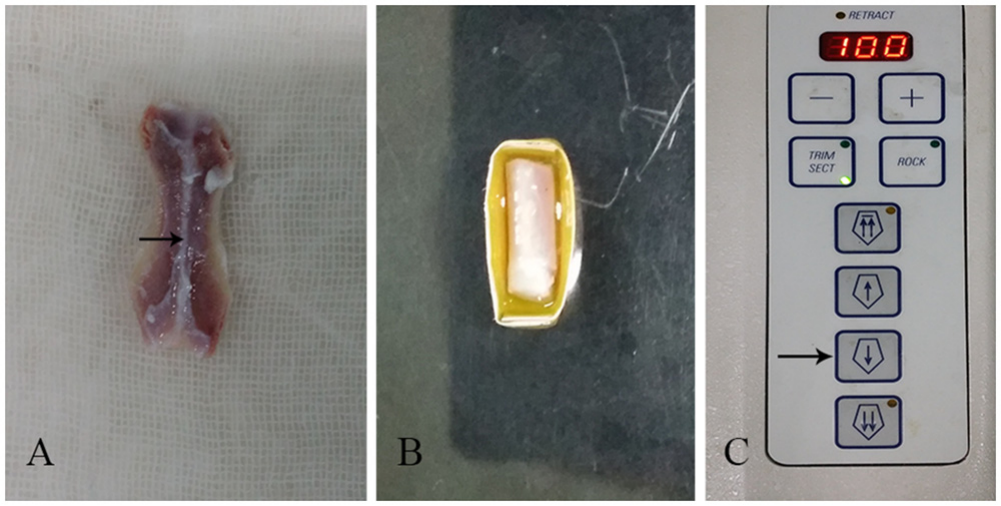
(A) The pubic symphysis fibrocartilage samples; (B) Embedding in the optimum cutting temperature (OCT) compound; (C) A single click representing a precession of 20 μm. 100 20 μm was achieved by 5 clicks for each layer.

After removing the soft tissue and bone, the fibrocartilage was trimmed into segments measuring approximately 10 mm × 3 mm × 2 mm, including part of the subchondral bone, which would be advantageous for the successful completion of the next freeze-sectioning step. The trimmed cartilage samples were fixed to the cutting base plate of a cryostat (Leica CM1950; Nussloch, Germany) with optimum cutting temperature (OCT) compound (polyvinyl alcohol and polyethylene glycol, Tissue-Tek1; Sakura Finetek USA, Inc., Torrance, CA, USA) and placed for 20 min at a presetting temperature of −22°C. The cartilage segments were then sliced to a thickness of 100 μm but left uncut at one end, which was achieved by using the near spin button with a single click representing a precession of 20 μm. Thus, a book-type fibrocartilage tissue scaffold was achieved by 5 clicks for each layer, and the thickness of each layer of the “book” was 100 μm. Ten layers were added to each “book” scaffold, and its final size was approximately 10 mm × 3 mm × 1 mm (Fig 1).

### Preparation of the acellular fibrocartilage scaffold

An improved decellularization method was developed according to published methods, which had previously been used to decellularize articular cartilage [16–18]. The sliced fibrocartilage samples were washed twice with phosphate-buffered saline (PBS) (3 × 30 min), followed by 3 freeze-thaw cycles (one cycle: freezing for 10 min in liquid nitrogen was followed by thawing for 10 min in a water bath at 37°C). Subsequently, the samples were immersed in 0.1% Triton X-100 with 1.5 M KCl for 12 h at 4°C with gentle agitation. They were then washed with 10 mM Tris buffer (pH 8.0) for 3 h, followed by another wash with PBS for 3 h. After these washes, the samples were digested in 0.25% trypsin (Genview, USA) for 24 h at 37°C with gentle agitation. Following a further 24-h wash in PBS, with rinsing repeated every 4 h during this period, the samples were incubated in nuclease solution (including 500 U/mL deoxyribonuclease I and 1 mg/mL ribonuclease A; Genview) for 12 h at 37°C with agitation to remove nuclear materials. Finally, the samples were repeatedly rinsed in PBS with agitation for 24 h, with rinsing repeated as described above.

### Histological analyses

The decellularized fibrocartilage scaffolds (n = 2 for each group) were placed flat onto filter papers to remove superfluous water from the samples’ surfaces. They were then fixed to the cutting base plate of the Cryostat Microtome System with OCT compound, where they were frozen at −22°C for 20 min. Subsequently, the samples were cut into 8-μm-thick sections in a direction perpendicular to the “book” and then placed onto glass slides. Before microscopic examination, sections were stained using hematoxylin and eosin (H&E) stain, Masson’s trichrome stain (KeyGEN Biotech, China), and 4,6-diamidino-2-phenylindole (DAPI) (Sigma-Aldrich, USA) to detect the cellular components and collagen fibrous structures.

### Scanning electron microscopy

Fibrocartilage “book” sheets with or without cells (n = 2 for each group) were rinsed with PBS and fixed overnight in 0.05% glutaraldehyde at 4°C. Following dehydration via a graded series of acetone, and replacement via a graded series of isoamyl acetate, samples were critical-point dried. Scaffolds were then coated with gold/palladium by using a sputter coater (EiKO-IB-5, Japan), and their surface morphology was visualized by using a scanning electron microscope (SEM) (Hitachi S-3400N, Japan).

### Quantification of residual DNA, collagen, and GAG in the ECM scaffolds

After the decellularization treatment, the samples (n = 8 for each group) were frozen overnight at −80°C. Subsequently, the samples were freeze-dried using a lyophilizer (SIM International Group, USA) at −70°C for 24 h. The freeze-dried samples were weighed and minced. According to the DNeasy Blood & Tissue protocol (Qiagen, Germany), 10 mg of lyophilized sample was digested with proteinase K (supplied with the kit) at 56°C for 3 h. The concentration of the purified DNA was measured by a spectrophotometer (MaestroNano, USA), and the quantity of DNA was calculated according to the DNA concentration. Standard DNA (Sigma-Aldrich) was used to make a standard curve. For the analysis of collagen, the Blyscan collagen assay kit (Biocolor, UK) was used. The lyophilized samples were digested in pepsin (Sigma-Aldrich) (1.0 mg/mL 0.5 M acetic acid) at 4°C for 48 h. The amount of collagen was measured using a Multiskan MK3 microplate reader (Thermo Scientific, USA). Fluorescence intensities were used to calculate the amount collagen in the scaffolds based on a standard curve produced by using standard collagen supplied with the kit. A Sircol GAG assay (Biocolor) was used to quantify the GAG that remained in the ECM scaffolds. The lyophilized samples were digested in 0.2 mg/mL papain (Sigma-Aldrich) in PBS (pH 6.4) at 65°C for 18 h. The amount of GAG was measured using the Multiskan MK3 microplate reader. Fluorescence intensities were also used to calculate the amount of GAG based on a standard curve produced using standard GAG supplied with the kit.

### Statistical analysis

The data are given as mean±standard deviation. One-way analysis of variance was used to determine the statistical significance between the different groups. Data were analyzed using SPSS17.0, and P < 0.05 was considered statistically significant.

## Results

### Characterization of the book-shape cartilage scaffold

**Fig 2:**
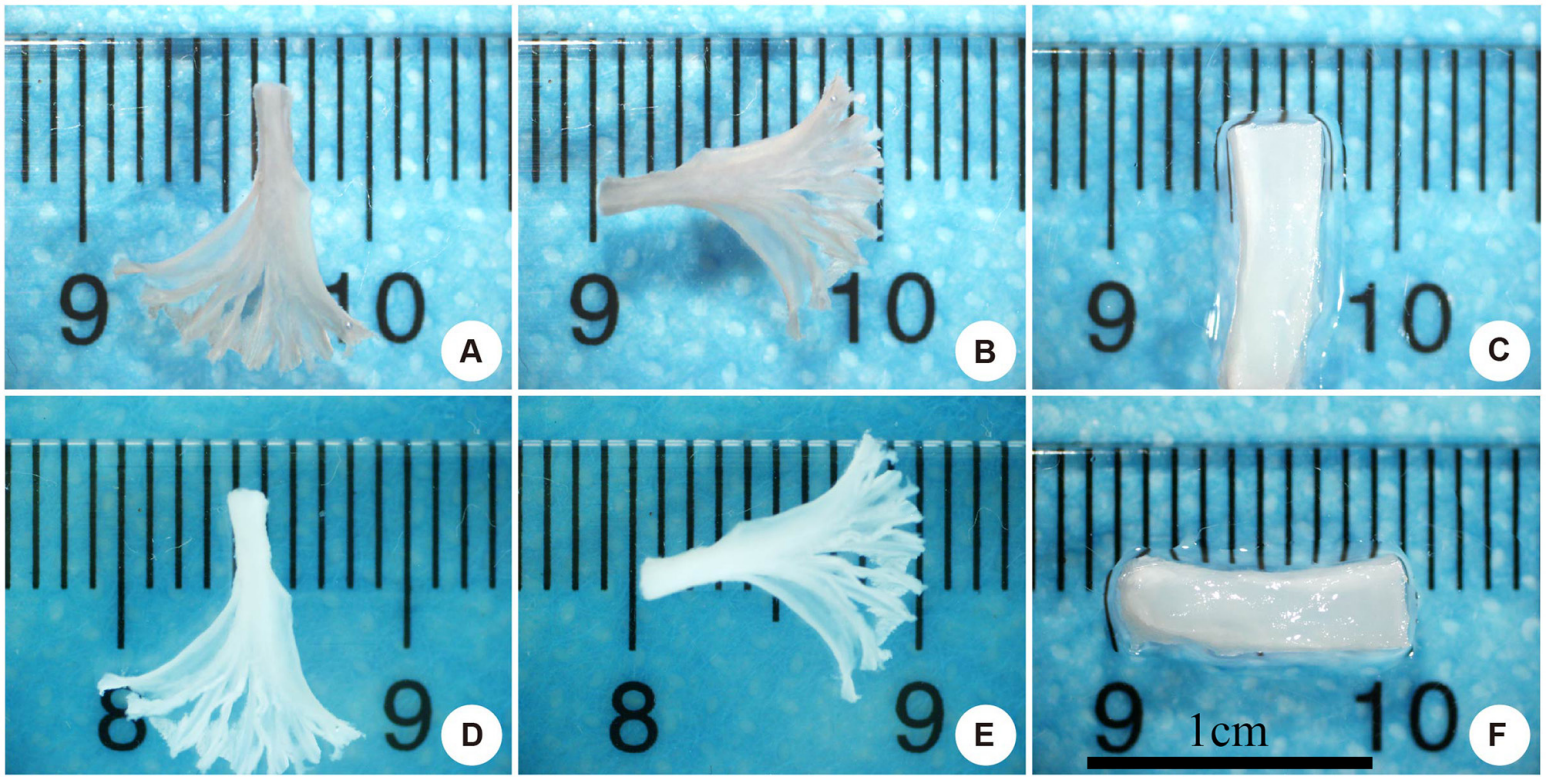
(A–C) The size of “book”-shape cartilage scaffolds before decellularization. (D–F) The size of decellularized “book” cartilage scaffolds.

The “book” fibrocartilage scaffolds were produced using freeze-sectioning. The size of the scaffold was 10 mm × 3 mm × 1 mm (Fig 2A–2C). The thickness of a layer was 100 μm. After decellularization, the scaffolds retained their original shape and size but were more transparent and white in color (Fig 2D–2F).

### Histological analyses

**Fig 3:**
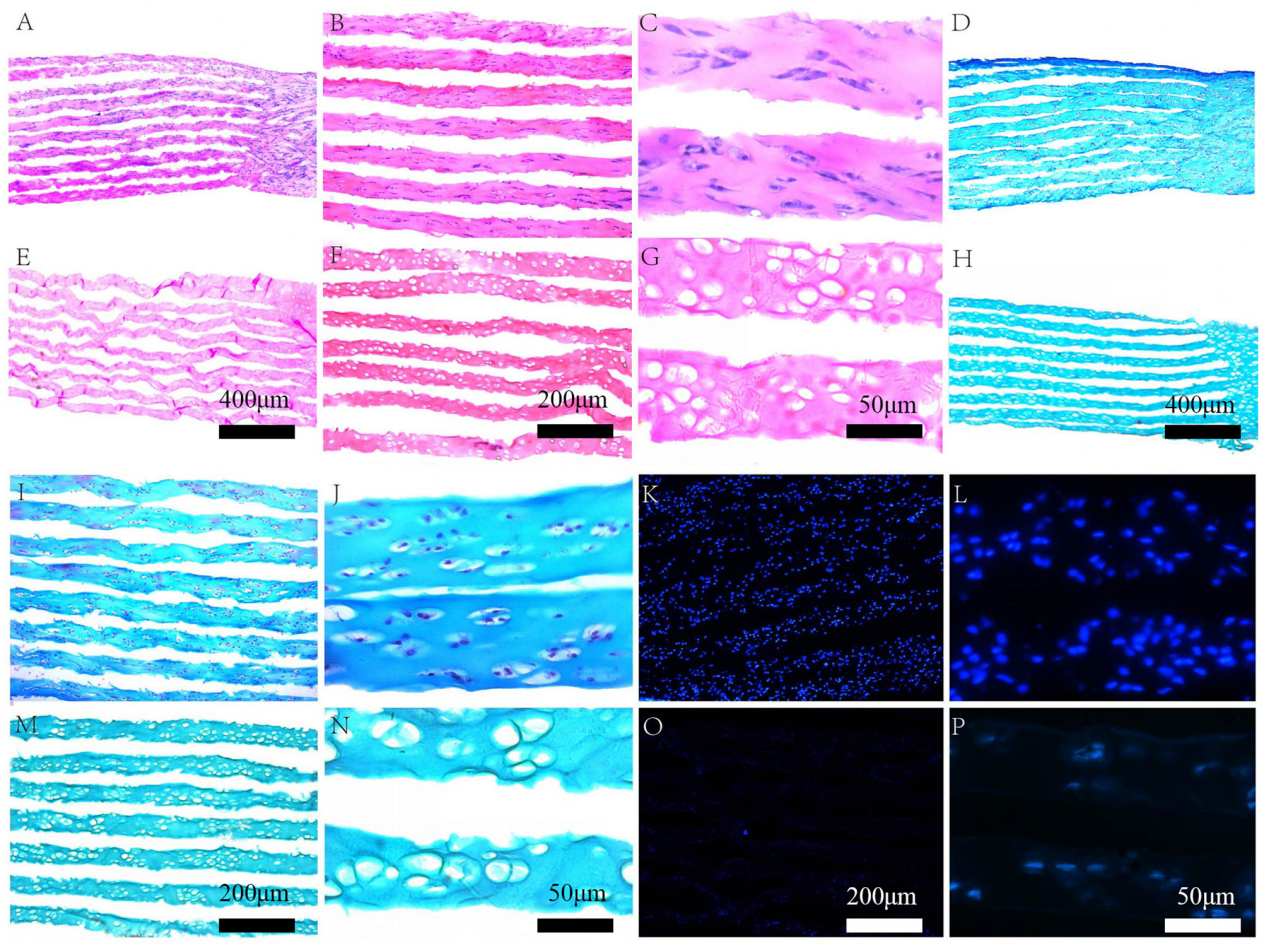
(A–C) Hematoxylin and eosin (H&E) staining of “book” cartilage scaffolds before decellularization (magnification: A, ×50; B, ×100; and C, ×400); (E–G) H&E staining of decellularized “book” cartilage scaffolds (magnification: E, ×50; F ×100; and G, ×400); (D, I, J) Masson’s trichrome staining of “book” cartilage scaffolds before decellularization (magnification: D, ×50; I, ×100; and J, ×400); (H, M, N) Masson’s trichrome staining of decellularized “book” cartilage scaffolds (magnification: H, ×50; M, ×100; and N, ×400); (K, L) 4ʹ,6-diamidino-2-phenylindole (DAPI) staining of “book” cartilage scaffolds before decellularization (magnification: K, ×100; L, ×400); (O, P) DAPI staining of decellularized “book” cartilage scaffolds (magnification: O, ×100; P, ×400 magnification).

H&E and Masson’s trichrome staining demonstrated that the cellular component was removed from the scaffolds following decellularization, while the native collagen structure was preserved. Furthermore, DAPI-positive cell nuclei were rarely observed after treatment (Fig 3A–3P).

### SEM analyses

**Fig 4:**
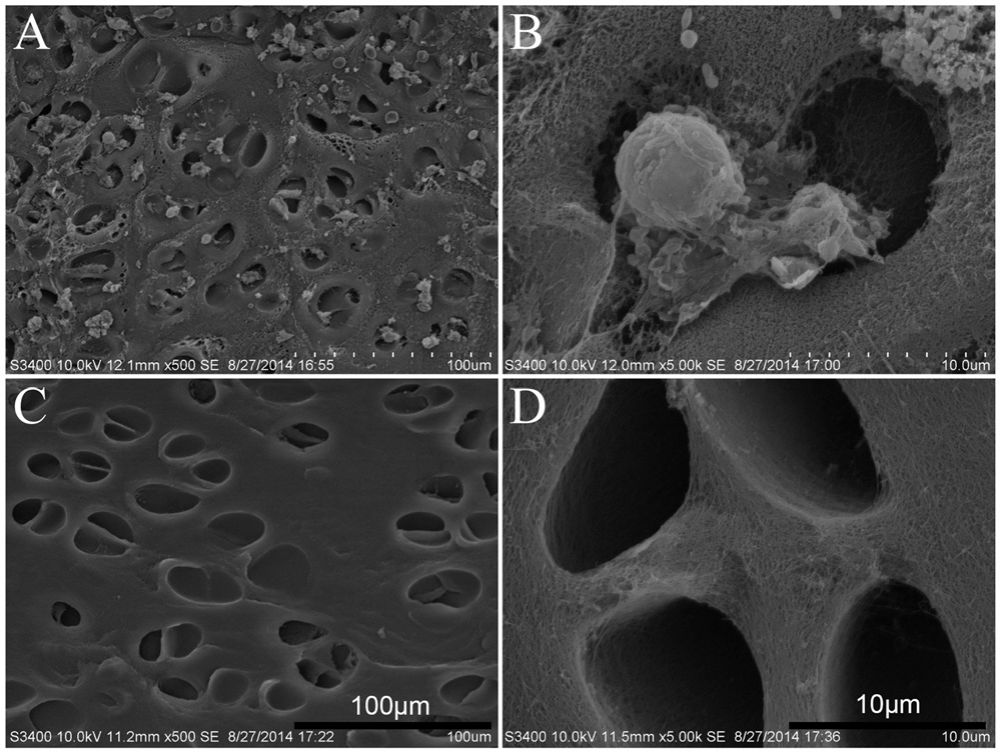
(A, B) Scanning electron microscopy (SEM) of “book” cartilage scaffolds before decellularization (A ×500 SE; B ×5000 SE); (C, D) SEM of decellularized “book” cartilage scaffolds (C ×500 SE; D ×5000 SE).

Using SEM, empty lacunar structures were observed in all decellularized scaffolds. High-magnification micrographs revealed that the characteristic cartilage lacuna and native collagen structure were well preserved after decellularization (Fig 4A–4D).

### Quantification of DNA, collagen, and GAG

**Fig 5:**
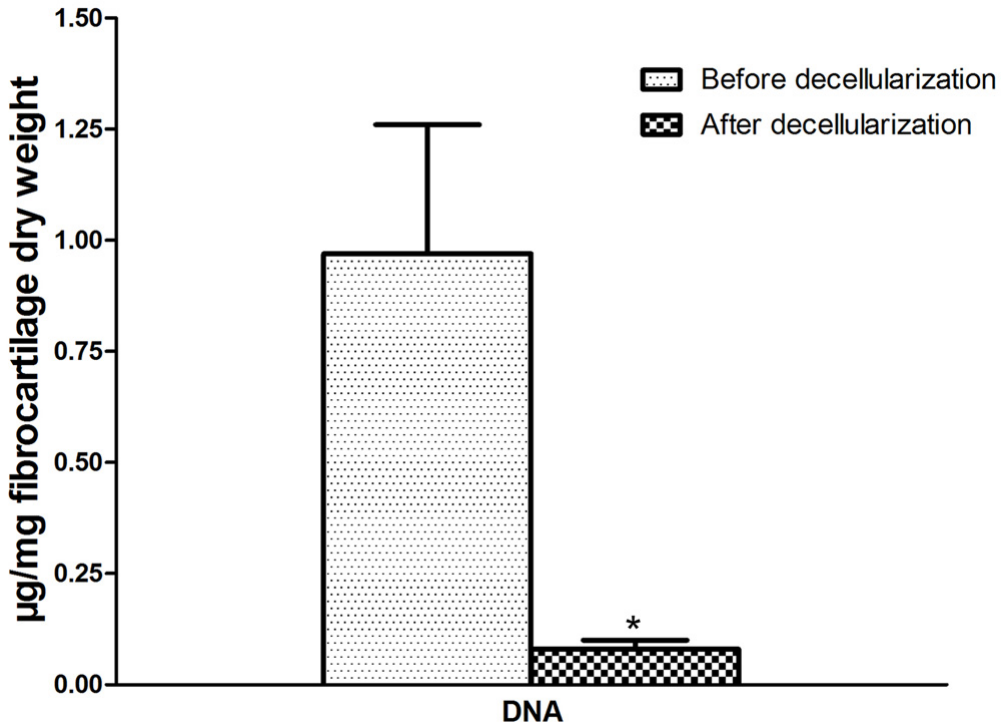
Quantification of DNA in fibrocartilage. DNA was measured at 0.97±0.29 μg/mg (fibrocartilage dry weight) before decellularization and 0.08±0.02 μg/mg after decellularization.

**Fig 6:**
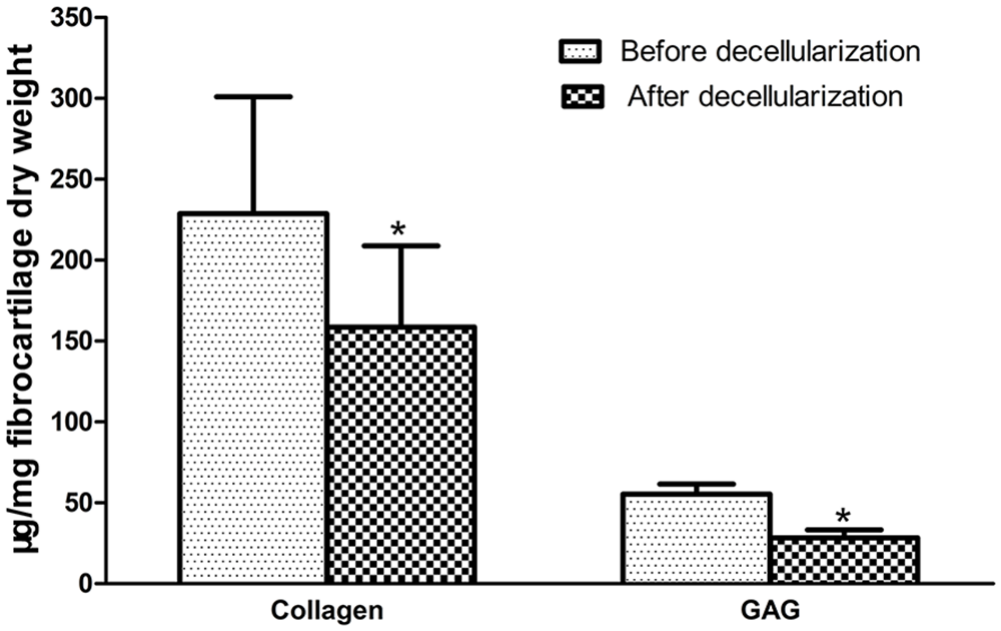
Quantification of collagen and GAG. The quantity of collagen was 228.68±72.32 μg/mg (dry weight) before decellularization and 158.70±50.17 μg/mg after decellularization. The quantity of GAG was 55.43±6.11 μg/mg (dry weight) before decellularization and 28.41±4.83 μg/mg after decellularization.

The quantity of DNA in the fibrocartilage samples was 0.97±0.29 μg/mg (fibrocartilage dry weight) before decellularization and 0.08±0.02 μg/mg after decellularization. The amount of DNA in the decellularized fibrocartilage ECM scaffolds was significantly lower than in the fresh fibrocartilage (P < 0.05). The quantity of collagen in the fibrocartilage samples was 228.68±72.32 μg/mg (dry weight) before decellularization and 158.70±50.17 μg/mg after decellularization (P < 0.05). This represented a 30.3% loss of collagen in the decellularized fibrocartilage ECM scaffolds compared with the fresh fibrocartilage. The quantity of GAG in the fibrocartilage samples was 55.43±6.11 μg/mg (dry weight) before decellularization and 28.41±4.83 μg/mg after decellularization (P < 0.05). Therefore, there was a 48.7% loss of GAG in decellularized ECM scaffolds compared with fresh samples (Figs 5 and 6).

## Discussion

Tendons and ligaments attach to bone through a transitional fibrocartilage zone that has complex biomechanical properties. This unique tissue structure is often not regenerated during the healing of an injury and/or surgical reattachment [19]. A natural, porous, easy-to-use, multilayered, decellularized “book” fibrocartilage scaffold was developed. This was achieved by developing fibrocartilage into a book-type decellularized bio-scaffold, a structure that was completely natural and had good biocompatibility. By engineering tissues, donor site morbidity can be avoided and large quantities of tissue with good histocompatibility can be produced [14]. Scaffolds play an important role in tissue engineering because they provide tissue-specific compositions that influence the attachment and growth of cells. Common musculoskeletal tissue engineering scaffolds are primarily divided into synthetic and natural biomaterials. The application of scaffolds made from synthetic biomaterials is somewhat limited by their inherent lack of biological activity [20, 21]. However, scaffolds made from natural biomaterials simulate the natural structure of biological tissue. Therefore, they may exhibit superior histocompatibility to synthetic scaffolds. In recent years, ECM-based scaffolds have shown great potential in tissue engineering [20]. Decellularized ECM from donor tissues has been used for constructing many complex tissues and organs [22–25]. Decellularization is an important process in the preparation of ECM scaffolds. By using chemical and physical processing, cell membranes and nuclei are disrupted, and the majority of cells and residual cellular components are removed from the tissues. This ensures that inflammatory and immune responses directed toward the scaffold are minimized, while the naturally occurring structure, organization, and biomechanical properties of the ECM are maintained [14]. Therefore, decellularized ECM scaffolds closely mimic the natural tissue environment and have better porosity and biocompatibility, as well as adequate mechanical strength, which promote cellular attachment, proliferation, and differentiation [14, 26]. The decellularized “book” bio-scaffold developed in this study, which was also found to mimic the natural tissue well, would be novel supplementary to the decellularized ECM scaffolds currently available.

It is difficult to remove all native cells due the dense nature of cartilage. Many methods have been developed to decellularize cartilage, and physical, chemical, and/or enzymatic techniques are usually used in combination. However, currently, there is no standard method for cartilage decellularization [14]. Consequently, in this study, a combination of physical, chemical, and enzymatic methods was used to decellularize the fibrocartilage scaffolds. Physical methods, which include thermal shock (freeze-thaw cycles), agitation in solution, physical factors, and manual disruption (pulverization or cutting to thin slices), can rupture cell membranes and facilitate the transport of decellularization solution to the cells and cellular material from the tissue [13, 14, 27]. Furthermore, by cutting tissues into particles or thin pieces, cells can easily be removed, while the natural composition of the ECM remains largely preserved [11, 15, 18]. Chemical treatments include detergents, solvents, acidic and alkaline solutions, and ionic solution. Sodium dodecyl sulfate, an ionic detergent, and Triton X-100, a nonionic detergent, are effective for the decellularization of cartilage tissue, but exposing tissues to these agents for too long can alter the mechanical properties of the cartilage ECM. It is often advantageous to use several chemicals in a series of short wash cycles to increase efficiency and reduce the time that tissue is exposed to any individual chemical [27]. DNases and RNases are also commonly used to remove nucleic acids from the material [14, 28, 29]. On the basis of the known efficacy of these existing techniques, the fibrocartilage in this study was first sliced into a “book” shape, a thin sheet structure with 100-μm-thick layers, and then decellularized by a combination of freeze-thaw cycles and treatments with Triton X-100, trypsin, and a nuclease, with continuous gentle agitation. Following this procedure, DNA quantification and histology analyses confirmed that cells were substantially removed from the scaffolds. Moreover, SEM analysis confirmed that the intrinsic ultrastructure of fibrocartilage tissue was well preserved. However, minimal nuclear material remained in the fibrocartilage at the end of the decellularization process. It is possible that this remaining material could result in mild host immune reaction, and this feature will be investigated in further in vivo study. A significant reduction in GAG and collagen content was also observed in fibrocartilage after decellularization, and this finding is consistent with the results of previous decellularization studies [24, 28].

Fibrocartilage is mainly distributed in intervertebral and articular discs and the pubic symphysis. The pubic symphysis is a unique joint consisting of a fibrocartilaginous disc sandwiched between the articular surfaces of the pubic bones. Histologically, the fibrocartilage at the tendon—bone interface is similar to the fibrocartilage—bone interface at the symphysis pubis [30]. Therefore, in this research, fibrocartilage of the pubic symphysis was used as the donor for tissue engineering. The results of the current study provide a strong foundation for the production of a new three-phased multilayered decellularized “book” scaffold, which will include bone, cartilage, and tendon, and into which mesenchymal stem cells can be implanted and cultured. In future research, the related molecular biological mechanisms of the scaffolds will be explored using in vitro and animal experiments.

This study had some limitations, which are as follows. The investigation was performed in vitro; therefore, the biocompatibility of the natural scaffold in tendon-to-bone healing in vivo remains unknown. Secondly, the mechanical properties of the scaffold were not tested, nor were the experiments conducted using human subjects. Finally, GAGs is one of the main components of cartilage and play an important role in maintaining the structure of cartilage. The content of GAG was reduced in the current study. It was unknown how the biological activity were affected by the reduced GAG content.

## Conclusions

In summary, a natural, porous, easy-to-use, multilayered, decellularized “book” fibrocartilage scaffold was developed in this ex vivo study. The acellular “book” scaffold represents an optimal carrier and effectively mimics the native environment and the structure of fibrocartilage. We expect its successful application in future tissue engineering, particularly for the reconstruction of the tendon-to-bone insertion.

## Supporting Information

S1 Checklist ARRIVE Guidelines Checklist. (PDF)
